# Magnitude of Low Birth Weight and Associated Factors among Newborns Delivered in Dangla Primary Hospital, Amhara Regional State, Northwest Ethiopia, 2017

**DOI:** 10.1155/2019/3587239

**Published:** 2019-03-03

**Authors:** Asmare Talie, Mekuanint Taddele, Mulunesh Alemayehu

**Affiliations:** ^1^Department of Midwifery, College of Health Sciences, Debre Markos University, Debre Markos, Ethiopia; ^2^Department of Public Health, College of Health Sciences, Debre Markos University, Debre Markos, Ethiopia

## Abstract

**Background:**

Low birth weight is defined as a live born infant weighs less than 2 500 g regardless of gestational age. Globally, the prevalence of low birth weight ranges from 3% to 15%. Birth weight plays an important role in infant mortality, morbidity, development, and future health. The prevalence of low birth weight in Ethiopia was estimated to be 14% which is one of the highest percentages in the world. So, the aim of this study is to assess magnitude and associated factors of low birth weight among newborns delivered at Dangla Primary Hospital, Amhara Region, Ethiopia.

**Methods:**

An institution-based cross-sectional study was conducted at Dangla Primary Hospital from September 27 to June 10, 2017. Systematic random sampling technique was used to select the 232 study participants. A structured and pretested questionnaire was used to collect data. Data quality was assured by pretesting, training, and frequent supervision. Descriptive statistics were performed for the descriptive part of the study. Binary and multiple logistic regression analyses were conducted to identify independent predictors of low birth weight. Those variables and p-value < 0.25 were included in the multivariable logistic regression for controlling the possible effect of confounders. Finally, variables which had significant association were identified on the basis of AOR, with 95%CI and with P-value <0.05.

**Results:**

Magnitude of low birth weight was 10.3 %. Previous history of low birth weight [AOR = 3.2, 95% CI: (1.13-9.9)], additional food intake during the last pregnancy [AOR = 5.0, 95% CI: (1.2-16.2)], and preterm delivery [AOR = 2.1, 95% CI: (3.1-19.2)] were independent predictors of low birth weight.

**Conclusion:**

Magnitude of low birth weight in Dangla Primary Hospital was high. So, strengthening counseling systems for women through quality antenatal care on advantage of additional food intake and previous bad obstetric outcome is necessary to alleviate the delivery of low birth weight neonates in the study area.

## 1. Background

Low birth weight is defined as birth weight of a live born infant less than 2500g irrespective of gestational age. It is a public health problem in developing countries especially in sub-Saharan Africa [1]. Around 20 million infants are born each year with <2500gm weight, accounting for 17% of all births in the developing world, out of which 6% are observed in industrialized countries and 21% in developing countries [1, 2].

Reasons of low birth weight are mainly linked with either infant/their mothers' side. In developed countries, predominant cause of LBW is preterm birth, whereas in developing countries, Intrauterine Growth Restriction (IUGR) is predominant cause of LBW [3]. Mothers who had multiple gestations had a higher risk of delivering LBW babies [4]. The physical environment, specific and nonspecific infections, also plays an important role in determining the infant's birth weight and future health status [5, 6]. Moreover, demographic risk factors such as young maternal age, prime gravid, low educational level, and poor maternal nutritional status before and during pregnancy are well recognized as risk factors for poor birth outcomes [7].

Concertinaing outcomes of low birth weight, the odds of developing chronic diseases such as hypertension, cardiovascular diseases, type II diabetes, metabolic syndrome, ischemic heart disease, decreased lung capacity, behavioral disorders, impaired physical, cognitive, and psychological function, and having long-term financial burden and chronic lung disease are higher among newborns weight <1500gm [8–12].

In general, birth weight is an important health status indicator of an infant and is a principal factor that determines the infant's physical, survival, and mental growth. It also indicates past and present health status of the mother [13, 14]. But, little attention is paid to birth weight improvement as a means of reducing child mortality in most developing countries including Ethiopia. It was approximated that every ten seconds an infant die of low birth weight related diseases. According to the WHO country cooperation strategy 2008–2011 report, the prevalence of low birth weight in Ethiopia was estimated to be 14% which was one of the highest percentages in the world [15]. Therefore, this study aims to assess low birth weight and its associated factors that will help as a base for other researchers, health care providers, and policy makers for further designing of strategic plan and intervening accordingly.

## 2. Methods and Materials

### 2.1. Study Area, Setting, and Period

The study was conducted at Dangla Primary Hospital, which is found in Awi Zone, Amhara region, Northwest Ethiopia. It is located in 72 km from South of Bahir Dar regional city. The hospital was established in 2015 as District hospital in Dangla Woreda, Awi Zone. It provides promotive, preventive, curative, and rehabilitative services for about 100,000 catchment population. At the time of its establishment, about 120 staffs were recruited, of them 66 were health professionals and the remaining were supportive staffs. Now the hospital has five wards, namely, medical (19 bed), surgical (21 beds), obstetrics, and gynecology ward (24 beds) and pediatrics (10 beds). Obstetrics and gynecology wards have separate labor, delivery, and postnatal care services and also have family planning, high risk maternity, antenatal care (ANC), and gynecological outpatient department (OPD) [16]. Currently the hospital serves around 34,200 populations. The study was conducted from September 27 to June 10, 2017.

### 2.2. Study Design and Population

An institutional-based cross-sectional study was conducted among sampled mothers who delivered in Dangla Primary Hospital during the study period and fulfill the inclusion criteria.

### 2.3. Sample Size and Sampling Techniques

Sample size was calculated by using Single population proportion formula by taking 17.4% from similar recent research performed in Gondar Referral Hospital, Northern Ethiopia [4]. By using the 95% CI and 5% marginal error (d)

(1) $$\begin{array}{c} \left(\;n=\frac{\left(Za/2\right)2p\left(1-p\right)}{d^{2}}\;\right), \end{array}$$

which gave sample size of 221. By adding 5% nonresponse rates the final sample size becomes 232.

### 2.4. Sampling Procedure

Systematic random sampling was employed to select study participants. According to Dangla Primary Hospital's delivery report, a total of 240 women delivered per month. Therefore, 232 study participants were selected by systematic random sampling technique. By taking the final sample size (n= 232), K was one. Thus, the study participants were selected. To get the initial study participant lottery method was used. Then, each study participant was selected using systematic random sampling technique. But, when the selected study participant did not fulfill the inclusion criteria, the next individual was included.

### 2.5. Data Collection Tool

Well-structured interviewer administered questionnaire was prepared. The questionnaire was prepared in English language and translated to local language Amharic and back to English to check consistency.

### 2.6. Data Quality Assurance

Pretesting was conducted in 5% of the sample size in Durbete Hospital before the actual data collection. A total of two days intensive training was given for all supervisors and data collectors. Double entry was done to minimize error.

### 2.7. Data Processing and Analysis

Data were checked for completeness and consistency and entered using Epi Data version 3.1 and then exported to SPSS version 22 for analysis. First descriptive analysis was carried out to determine the magnitude of LBW. Bivariate analysis was used primarily to check which variables had associated with the dependent variable. Those variables and p-value < 0.25 in the bivariate analysis were included in the multivariate logistic regression for controlling the possible effect of confounders. Finally, variables which had significant association were identified on the basis of AOR, with 95%CI and with P-value ≤0.05.

### 2.8. Ethical Consideration

Ethical permission was obtained from the ethical review committee of the College of Health Sciences, Debre Markos University. After obtaining ethical permission, written letter was sent to Awi Zonal health department to get permission. Then permission letter was obtained from Awi Zonal Health Department and Dangla Town Administration Health Office and respective bodies to conduct the study. Also verbal informed consent was obtained from each study participant.

## 3. Results

### 3.1. Sociodemographic Characteristics

Of the total 232 mothers, 217 of them were participated in the study with a response rate of 94 %. The mean age of the respondent was 26.6 years. More than half (137, 59.1%) of the respondents were orthodox Christian follower, 188 (81.1%) can read and write, and 20 (8.6) respondents were divorced. The detailed sociodemographic characteristics of the participant are described in Table 1.

### 3.2. Magnitude of Low Birth Weight

In this study, the magnitude of low birth weight was 10.3 %. Among low birth weight neonates, 22 (9.4%) were between 1500 and 2499gm. The mean birth weight of the neonate was 3.14 kg. Fifty-two (22.4%) of the neonates were delivered before 37 wks of gestation. Of the respondents, 136 (58.6%) of mothers delivered between 37 and 42 weeks of gestation (Table 2). Based on nutritional status of women in the last pregnancy, 183 (78.9 %) of women had nutritional counseling and 151 (65.1%) had taken additional diets during pregnancy.

### 3.3. Reproductive Characteristics

**Figure 1:**
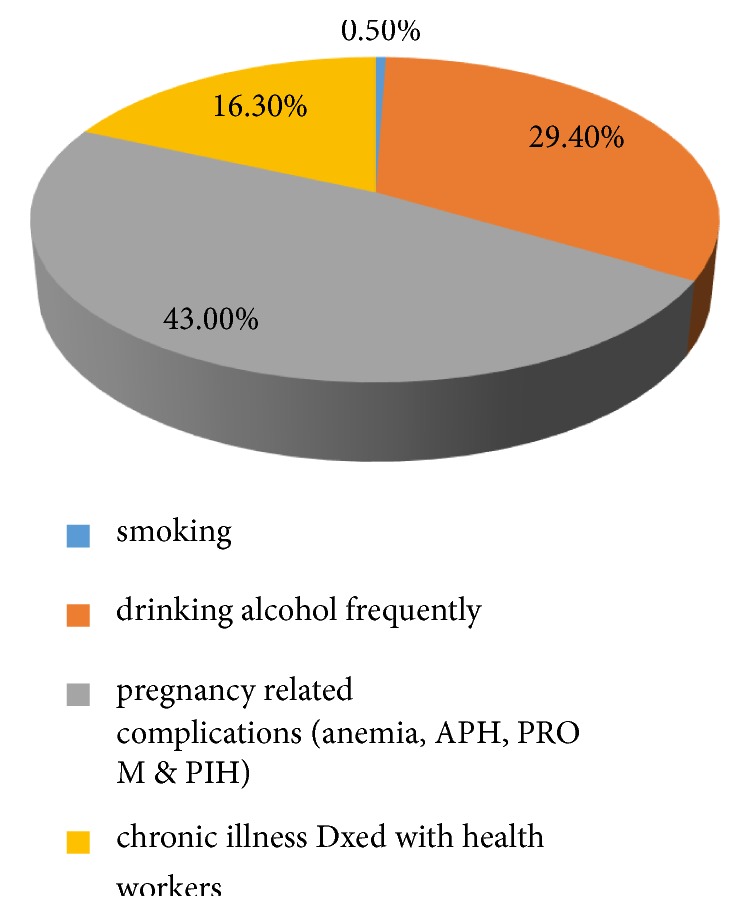
Health status of mothers who delivered at Dangla Primary Hospital, Awi Zone, Amhara Region, Northwest Ethiopia, 2017 (n=232).

Among the respondents, 37 (15%) of them were reported previous abortion, 32 (13.8%) of the clients had a low birth weight in the previous pregnancy, and 185 (79.7 %) of the client have ante natal care follow-up, when we see the gravidity of respondents, gravida one 67 (28.9%), gravid two 67 (28.9%), gravida three, and above 98 (42.25%) (Figure 1).

### 3.4. Factors Associated with Low Birth Weight

Variables considered for multivariable logistic regression analysis were those with a p-value<0.2 in bivariable analysis and those significantly associated with bivariable analysis were previous history of low birth weight, no ANC follow-up, women having no additional food intake during last pregnancy, pregnancy complication, gestational age, and infant sex.

After controlling confounding variables using multiple logistic regressions, low birth weight of the previous pregnancy, gestational age<37 weeks, and women who have no additional food intake during pregnancy were independent predictors of LBW. Women who had previous history of low birth weight had 3.2 times higher odds ratio of delivered low birth weight baby than their counterparts [AOR = 3.2, 95% CI: (1.1-9.9)]. Those pregnant women who did not have additional food intake had 5.0 times higher odds ratio of delivered low birth weight neonates than those who had additional food intake during the current pregnancy [AOR = 5.0, 95% CI: (1.2-16.2)] and pregnant women who delivered before 37 weeks of gestational age had 2.14 times higher odds ratio of delivered LBW neonates than those delivered at term [AOR = 2.1, 95% CI: (3.1-19.2)] (Table 3).

## 4. Discussion

This study was aimed at determining the magnitude of low birth weight and associated factors in Dangla Primary Hospital. The finding of this study indicated that 10.3% of neonates were born with low birth weight. The finding is in line with the studies conducted in India (11 %) [2] and institutional based retrospective study conducted in Korowai District Hospital, Tanzania (9.1%) [17], whereas this finding is lower than the global prevalence (17%) [1] and study conducted in Gondar University Hospital, Northwest Ethiopia (17.4%) [4] and in Jimma Zone, Southwest Ethiopia (22.5%) [17]. These differences might be explained due to variation in study setup, population difference, study time, and study design.

This study did not find any significant association between low birth weight and sociodemographic factors including maternal age, residence, educational status, monthly income, religion, marital status, and ethnicity. Several studies have shown that sociodemographic factors can influence low birth weight either directly or indirectly [18].

In this study, previous history of low birth weight explained a significant association with low birth weight. Those women having previous history of LBW had higher odds to have delivery of LBW neonates than women who did not have previous history. This result is similar to the study conducted in Nigeria [19] and Japan [14].

The present study revealed that there is a statistically significant association between additional food intake during last pregnancy and occurrence of LBW. The odds of women who did not have additional food intake in their last pregnancy had get more chance of delivering LBW neonates compared to those mothers who had balanced diet. Again the study conducted in Pakistan [20], Japan [14], and Addis Ababa [19] proved this.

In addition, this study showed that LBW was significantly associated with gestational age. The odds of women who gave birth before 37 weeks of gestational age in their last pregnancy had get increased chance of LBW neonates compared to those mothers who delivered at term pregnancy.

The finding is similar to studies conducted in Mekele p-value=0.0001 [8], Gondar p-value<0.005 [10] , and Jimma p-value<0.005 [5]. 

## 5. Conclusion

The magnitude of low birth weight in Dangla primary hospital was high. Low birth weight in the previous pregnancy, gestational age<37 weeks, and women having no additional food intake during the current pregnancy showed significant association with LBW neonates. Based on the results the following recommendations are forwarded:

*(I) Health Institutions. *To reduce LBW neonate health care providers need to work to early detect and manage risk factors that cause preterm delivery of the newborn. It is advisable that those health care providers need to work in the community on the importance of balanced diet during pregnancy and the consequence of previous pregnancy bad outcomes to the current pregnancy through focused antenatal care on the prevention of LBW newborns.

*(II) Couples. *As pregnancy suspected, it is better to early come to health institutions and get counseling about importance of nutrition and the factors which lead to preterm birth to prevent LBW newborns.

*(III) Researchers. *It is better to do further community based studies.

## Figures and Tables

**Table 1:** Sociodemographic characteristics of women delivered at Dangla Primary Hospital, Awi Zone, Amhara Region, Northwest Ethiopia, 2017 (n=232).

Variables	Category	Frequency (N)	Percentage (%)
Age	15-25	78	33.6
	26-36	101	43.5
	37-47	40	17.3
	>47	13	5.6
Religion	Muslim	54	23.3
	Orthodox	137	59.1
	Protestant	33	14.2
	Catholic	8	3.4
Marital status	Married	181	78
	Single	21	9.1
	Divorced	20	8.6
	Widowed	10	4.3
Educational status	Can't read and write	44	18.9
	Can read and write	188	81.1
Monthly income	<620	15	6.5
	621-1500	30	12.9
	1501-3000	66	28.4
	>3001	121	52.2
Occupation	Housewife	57	24.6
	Merchant	44	19
	G /employed	47	20.3
	Daily labor	10	4.2
	Farmer	74	31.9
Ethnicity	Oromo	14	6
	Tigre	8	3.4
	Amhara	204	87
	Others	6	3.6
Residence	Urban	122	52.6
	Rural	110	47.4

**Table 2:** Labor and delivery characteristics of mothers delivered at Dangla Primary Hospital, Awi Zone, Amhara Region, Northwest Ethiopia, 2017 (n=232).

Variables	Category	Frequency (n)	Percentage (%)
Gestational age	<37wks	52	22.4
	37-42wks	136	58.6
	>42wks.	44	19
Gender of the neonate	Male	118	50.9
	Female	114	49.1
Birth weight	>=2.5kg	208	89.6
	1.5-2.499	22	9.50
	1-1.5	2	0.9

**Table 3:** Multivariable analysis results, factors affecting LBW among neonates delivered in Dangla Primary Hospital, Awi Zone, Amhara Region, Northwest Ethiopia, 2017 (n=232).

Variable	Category	COR (95%CI)	AOR (95% C.I.)	P- value
ANC follow-up	Yes	1		
	No	5.7(1.2-4.5)	1(0.5-28.9)	0.06
Additional diet	Yes	1		
	No	3.1(5.0-6.2)	5.03(1.8-18.8)	*0.002* ^*∗*^
Previous history of LBW	Yes	1.5(1.8-3.0)	3.21(1.5-14.2)	*0.001* ^*∗*^
	No	1		
Pregnancy complication	Yes	2.1(3.4-6.3)	2(0.1-7.6)	0.09
	No	1		
Gestational age	<37 wks.	4.8(1.2-2.3)	2.1(1.3-10.4)	*0.004* ^*∗*^
	>37 wks.	1		
Infant sex	Male	1		
	Female	1.8(4.6-5.2)	3(0.2-4.7)	0.08

NB: ^*∗*^significant association, P <0.05.

AOR=adjusted odds ratio.

## Data Availability

The data used to support the findings of this study are available from the corresponding author upon request
